# Distinguished Visitors

**Published:** 1905-09

**Authors:** 


DISTINGUISHED VISITORS.—I take pleasure in announcing that during the month of October next—the exact date will be published in the newspapers—Dr. Joseph Price, of Philadelphia, will visit the profession of Texas and will make his headquarters at Dallas, where elaborate preparations are being made to entertain him. He is the especially invited guest of the Dallas County Medical Society, which hopes that the profession throughout the State will co-operate to make the coming of this distinguished surgeon pleasant and profitable to all concerned. The probability is that Dr. Price will be accompanied by the eminent surgeons Dr. John Deaver, of Philadelphia, and Dr. Lewis McMurtry, of Louisville. These gentlemen are intimate friends of Dr. Price, and it is certain almost that they will come with him to Texas. The profession of Texas is to be congratulated upon the pleasing prospect of doing a great honor to three of the most progressive surgeons of the period, and who will come a great distance to meet their many friends in Texas and demonstrate advanced operative methods in the fields of general and abdominal surgery. Clinics for operations will be held at several hospitals in Dallas, and a rare opportunity there afforded physicians for securing and witnessing the skill of three of the most famous exemplifiers of modern surgery well known wherever the surgical art is practiced. It is believed that the profession will give them an overwhelming reception that will prove a notable event in the history of our State. Their stay in Dallas will be limited to two days only.

